# When the Mind Fails: A Mysterious Case of Concurrent Neurosyphilis, Herpes Simplex Virus Encephalitis, and Suspected Autoimmune Encephalitis

**DOI:** 10.7759/cureus.72415

**Published:** 2024-10-26

**Authors:** Nava R Sharma, Madalasa Pokhrel, Prabal KC, Sumitra Paudel, Prakriti Lamichhane, Marlon E Rivera Boadla, Barbara Alvarez

**Affiliations:** 1 Internal Medicine, Maimonides Medical Center, Brooklyn, USA; 2 Medicine, Manipal College of Medical Science, Pokhara, NPL; 3 Infectious Disease, Maimonides Medical Center, Brooklyn, USA; 4 Public Health, The University of Southern Mississippi, Hattiesburg, USA; 5 Research, Maimonides Medical Center, Brooklyn, USA; 6 Pathology, Kist Medical College, Lalitpur, NPL

**Keywords:** anti-nmda receptor encephalitis, autoimmune encephalitis, encephalitis, hsv encephalitis, late neurosyphilis

## Abstract

Herpes simplex virus (HSV) encephalitis is the most common cause of sporadic viral encephalitis and is associated with significant morbidity and mortality if not promptly recognized and treated. Neurosyphilis is now rare due to the widespread use of antibiotics. This case report discusses a 65-year-old man with no notable past medical history who presented to the emergency department with acute shortness of breath, choking, and altered mental status, following six months of cognitive and behavioral decline. Initial evaluations revealed fever and hypoxia, necessitating urgent intubation. While most laboratory tests were unremarkable, cerebrospinal fluid (CSF) analysis demonstrated positivity for the venereal disease research laboratory (VDRL) test, HSV-1, and N-methyl-D-aspartate (NMDA) receptor antibodies, indicating the presence of neurosyphilis, HSV encephalitis, and questionable autoimmune encephalitis. Imaging studies showed chronic microvascular disease without significant lesions. The interplay of infectious and autoimmune processes complicated the clinical picture, emphasizing the need for thorough diagnostic evaluation. Prompt treatment with acyclovir for HSV encephalitis and penicillin for neurosyphilis was critical. This case underscores the necessity of considering multiple etiologies in patients with rapid cognitive decline, highlighting the importance of timely recognition and appropriate management in complex clinical scenarios.

## Introduction

Herpes simplex virus (HSV) encephalitis is the most prevalent cause of sporadic viral encephalitis, often leading to significant morbidity and mortality if not promptly recognized and treated [1]. It typically presents with a constellation of symptoms including altered mental status, fever, seizures, and focal neurological deficits [1,2]. Magnetic resonance imaging (MRI) typically shows hyperintense lesions in the temporal lobes, insular cortex, and limbic system on T2-weighted and fluid-attenuated inversion recovery (FLAIR) sequences in HSV encephalitis, often with associated edema and hemorrhage [1,2]. Neurosyphilis, a rare yet serious complication of untreated syphilis, can manifest with a diverse range of neurological symptoms, including cognitive decline and psychiatric disturbances [3]. Autoimmune encephalitis, particularly N-methyl-d-aspartate (NMDA) receptor encephalitis, has emerged as a critical area of focus due to its association with viral infections and underlying malignancies [4]. The concurrent occurrence of HSV encephalitis, neurosyphilis, and autoimmune encephalitis is exceptionally rare, making this case particularly significant in highlighting the complexities of differential diagnosis in patients with rapid cognitive decline.

A 65-year-old man with a six-month history of memory loss, tremors, and behavioral changes presented with acute shortness of breath and altered mental status, with cerebrospinal fluid (CSF) positive for venereal disease research laboratory (VDRL), HSV-1, and NMDA receptor antibodies. This case report is important as it underscores the complexities of diagnosing and managing patients with rapid cognitive decline and neurological dysfunction.

## Case presentation

A 65-year-old man with no significant past medical history presented to the emergency department (ED) with acute shortness of breath, choking, and altered mental status, following a six-month history of progressive cognitive and behavioral decline. His family reported that he had been well until approximately six months prior, when he began experiencing memory issues, tremors, and forgetfulness at work. Over the next few months, co-workers noticed abnormal behavior, including a random shouting episode, forgetfulness, and aggressive behavior. Following this, the family observed further behavioral changes, such as increased aggression, disorientation, and periods of not recognizing familiar faces. Despite a psychiatric evaluation that resulted in a diagnosis of schizophrenia and subsequent treatment, the patient's symptoms did not improve.

In the weeks leading up to his ED visit, the patient's condition worsened significantly. He developed gait disturbances, had difficulty eating, and exhibited a marked decline in cognitive function, including periods of not recognizing his own name. Additionally, he developed a fever and cough that did not improve. In the days preceding his presentation, the patient became averse to food, experienced vomiting while being fed, and ultimately developed acute shortness of breath, prompting his family to seek emergency care.

Upon arrival at the ED, the patient was febrile and found to be desaturating to 80%, necessitating urgent intubation. He was started on empiric antibiotic therapy (ceftriaxone and doxycycline) for community-acquired pneumonia coverage. Laboratory tests, including a complete blood count, liver function tests, and a basic metabolic panel, were unremarkable, as shown in Table 1 below.

**Table 1:** The initial laboratory results were unremarkable, except for a positive RPR. CBC: complete blood count; WBC: white blood cells; Hgb: hemoglobin; Hct: hematocrit; Plt: platelets; BMP: basic metabolic panel; Na: sodium; K: potassium; Cl: chloride; CO2: carbon dioxide; BUN: blood urea nitrogen; Cr: creatinine; Glu: glucose; Mg: magnesium; Ca: calcium; P: phosphorus; AG: anion gap; AST: aspartate aminotransferase; ALT: alanine aminotransferase; NH₃: ammonia; HbA1C: hemoglobin A1C; ESR: erythrocyte sedimentation rate; CRP: C-reactive protein; CPK: creatine phosphokinase; LDH: lactate dehydrogenase; RPR: rapid plasma reagin; EBV: Epstein-Barr virus; HIV: human immunodeficiency virus; CMV: cytomegalovirus; HSV: herpes simplex virus

Test	Result	Normal reference range
CBC		
WBC (×10^9^/L)	8.9	4-10
Hgb (g/dL)	14.8	13-17 (male)
Hct (%)	45.6	40-52 (male)
Plt (×10^9^/L)	193	150-450
BMP		
Na (mmol/L)	147	135-145
K (mmol/L)	4.1	3.5-5
Cl (mmol/L)	106	98-106
CO2 (mmol/L)	30	22-30
BUN (mg/dL)	26	7-20
Cr (mg/dL)	0.9	0.6-1.2
Glu (mg/dL)	122	70-126
Mg (mg/dL)	1.9	1.7-2.2
Ca (mg/mL)	9	8.5-10.5
P (mg/dL)	3.5	2.5-4.5
AG (mmol/L)	11	8-12
AST (U/L)	48	10-40
ALT (U/L)	51	7-56
NH₃	16	15-56 µmol/L
HbA1C	6%	4-5.6%
Vitamin B12 (pg/mL)	398	200-900
Folate (ng/mL)	7	>6.6
Troponin	0.01	<0.04 ng/mL
ESR (mm/hr)	53	0-20 (male)
CRP (mg/L)	3.28	<3
CPK (U/L)	305	59-367
LDH (U/L)	281	108-199
RPR	1:16	Negative
Hepatitis A IgG	Positive	Negative
Hepatitis A IgM	Negative	Negative
Hepatitis B surface antigen	Negative	Negative
Hepatitis B immunity (anti-HBs)	Positive	Positive (immune status)
Hepatitis C antibody	Negative	Negative
EBV antibody	Negative	Negative
HIV test	Negative	Negative
CMV test	Negative	Negative
HSV test	Negative	Negative

The patient was admitted to the medical intensive care unit with acute hypoxic respiratory failure, likely secondary to aspiration pneumonitis or pneumonia. Neurology was consulted to further evaluate the cognitive decline and tremors. A septic workup was initiated, including chest X-ray, urine microscopy, blood culture, respiratory culture, and respiratory viral panel. The patient remained on mechanical ventilation throughout this initial evaluation.

Neurological examination revealed a non-responsive patient who opened his eyes spontaneously but showed no movement in response to noxious stimuli. An electroencephalogram (EEG) demonstrated moderate diffuse cerebral slowing without epileptiform activity, suggesting moderate cerebral dysfunction of non-specific etiology. A computed tomography (CT) scan of the head was unremarkable, revealing only mild chronic microvascular disease.

A lumbar puncture was performed, and CSF analysis revealed elevated white blood cell (WBC) counts (38/µL) with a lymphocytic predominance (92%), consistent with central nervous system inflammation or infection. Notably, the CSF VDRL was positive (1:2), raising suspicion of neurosyphilis. CSF HSV-1 PCR returned positive, indicating HSV encephalitis, and acyclovir therapy was initiated. The CSF findings are tabulated below in Table 2.

**Table 2:** Initial CSF finding and repeat CSF finding done in three weeks. CSF: cerebrospinal fluid; WBC: white blood cell; RBC: red blood cell; VDRL: venereal disease research laboratory; NMO: neuromyelitis optica; MOG: myelin oligodendrocyte glycoprotein; OCBs: oligoclonal bands; t-tau: total tau; RT-QuIC: real-time quaking-induced conversion; NMDA: N-methyl-D-aspartate; N/A: not available

Test	Initial lumbar puncture	Repeat lumbar puncture	Normal reference range
CSF WBC	38 cells/µL (lymph predominant)	15 cells/µL (100% lymphocytes)	0-5 cells/µL
CSF RBC	36 cells/µL	1 cells/µL	0-5 cells/µL
CSF protein	86 mg/dL	46 mg/dL	15-45 mg/dL
CSF glucose	89 mg/dL	73 mg/dL	40-70 mg/dL
CSF VDRL	Low-positive (1:2)	Negative	Negative
CSF herpes simplex 1	Positive	Negative	Negative
CSF gram stain/culture/fungal	Negative	Negative	Negative
CSF *Cryptococcus* antigen	Negative	Negative	Negative
NMO/MOG antibodies	Negative	N/A	Negative
OCBs	Positive (>5 unique OCBs)	N/A	Absent
CSF Biofire	Negative	N/A	Negative
CSF cytology	Negative for malignant cells	N/A	Negative
14-3-3 protein	6315 AU/mL	N/A	<173-1999 AU/mL
t-tau protein	817 pg/mL	N/A	0-1149 pg/mL
RT-QuIC	Negative	N/A	Negative
NMDA-R Ab CBA, CSF	Positive	N/A	Negative

Testing for paraneoplastic and autoimmune etiologies revealed positive NMDA receptor antibodies, supporting a diagnosis of autoimmune encephalitis. The Mayo Clinic CSF Laboratories panel was negative except for NMDA-R Ab CBA, CSF as shown in Table 3 below.

**Table 3:** Mayo Clinic CSF autoimmune encephalitis and paraneoplastic antibody panel result. IgLON5: immunoglobulin LON5; LGI1: leucine-rich glioma inactivated 1; mGluR1: metabotropic glutamate receptor 1; NIF: neurofilament; PCA-Tr: Purkinje cell cytoplasmic autoantibody type Tr; PCA-1: Purkinje cell cytoplasmic autoantibody type 1; PCA-2: Purkinje cell cytoplasmic autoantibody type 2; NMDA-R: N-methyl-D-aspartate receptor; AMPA-R: alpha-amino-3-hydroxy-5-methyl-4-isoxazolepropionic acid receptor; AGNA-1: anti-glial nuclear antibody 1; ANNA-1: anti-neuronal nuclear antibody 1; ANNA-2: anti-neuronal nuclear antibody 2; ANNA-3: anti-neuronal nuclear antibody 3; CASPR2-IgG: contactin-associated protein-like 2 IgG; CRMP-5-IgG: collapsin response mediator protein 5 IgG; DPPX: dipeptidyl peptidase-like protein 6; GABA-B-R: gamma-aminobutyric acid receptor type B; GAD65: glutamic acid decarboxylase 65; GFAP: glial fibrillary acidic protein; CSF: cerebrospinal fluid

Result name	Result	Reference value
IgLON5 IFA, CSF	Negative	Negative
LGI1-IgG CBA, CSF	Negative	Negative
mGluR1 Ab IFA, CSF	Negative	Negative
Neurochondrin IFA, CSF	Negative	Negative
NIF IFA, CSF	Negative	Negative
PCA-Tr, CSF	Negative	Negative
PCA-1, CSF	Negative	Negative
PCA-2, CSF	Negative	Negative
Septin-7 IFA, CSF	Negative	Negative
NMDA-R Ab CBA, CSF	Positive	Negative
AMPA-R Ab CBA, CSF	Negative	Negative
Amphiphysin Ab, CSF	Negative	Negative
AGNA-1, CSF	Negative	Negative
ANNA-1, CSF	Negative	Negative
ANNA-2, CSF	Negative	Negative
ANNA-3, CSF	Negative	Negative
CASPR2-IgG CBA, CSF	Negative	Negative
CRMP-5-IgG, CSF	Negative	Negative
DPPX Ab IFA, CSF	Negative	Negative
GABA-B-R Ab CBA, CSF	Negative	Negative
GAD65 Ab Assay, CSF	0.00	≤0.02
GFAP IFA, CSF	Negative	Negative

**Figure 1:**
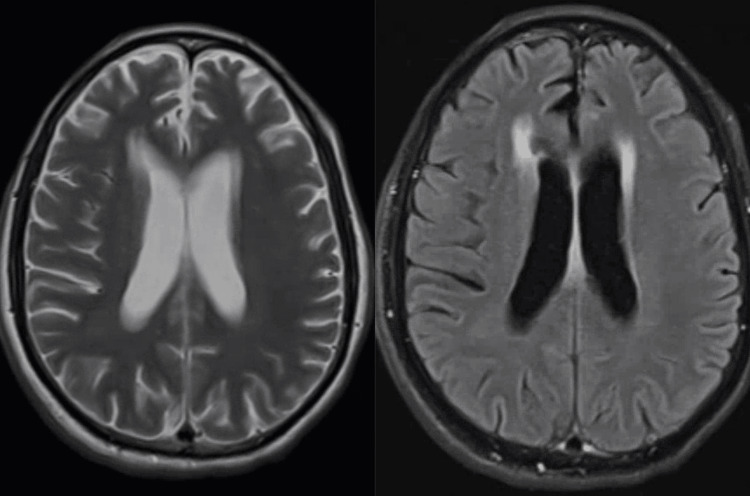
Axial T2/FLAIR image above demonstrates a small, ill-defined hyperintensity in the right posterior frontal subcortical white matter, consistent with gliosis related to chronic microvascular disease. FLAIR: fluid-attenuated inversion recovery

An MRI of the brain showed mild to moderate chronic microvascular disease and some ill-defined FLAIR hyperintensity in the frontal lobes, presumed to represent gliosis, as shown in Figure 1 below. No abnormal mass or lesions were reported.

The patient received intravenous acyclovir for HSV encephalitis and a 14-day course of penicillin for neurosyphilis. Repeat blood and urine cultures remained negative. A second lumbar puncture showed a reduction in CSF WBC count (15/µL) but persistently elevated lymphocytes. After completing his antimicrobial course, the patient was evaluated for discharge. His respiratory status later improved, and he was successfully extubated to supplemental oxygen via nasal cannula. He remained afebrile afterward. However, his cognitive status remained poor; although awake, he was unable to follow commands.

The patient was discharged with plans for outpatient neurology follow-up after 32 days of hospital admission and close monitoring of cognitive status, including a repeat MRI. The patient's family was educated about the multifactorial nature of his illness, encompassing both infectious and autoimmune components, and they were referred for further outpatient management.

## Discussion

A comprehensive differential diagnosis for this case includes autoimmune encephalitis, which could explain the rapid cognitive decline and behavioral changes; HSV encephalitis, supported by the positive CSF HSV-1 PCR; and neurosyphilis, indicated by the weak positive CSF VDRL result. Frontotemporal dementia (FTD) is another possibility, given the significant personality changes and cognitive dysfunction. Additionally, psychiatric illnesses such as acute psychosis or schizophrenia may account for the disorganized behavior observed. Creutzfeldt-Jakob disease (CJD) remains a concern due to the rapid progression of symptoms, although negative real-time quaking-induced conversion (RT-QuIC) testing reduces its likelihood despite elevated 14-3-3 protein levels. Other infectious processes, including various viral or bacterial encephalitides, and neoplasms, particularly paraneoplastic syndromes, were considered, although CT imaging of the chest, abdomen, and pelvis was unremarkable.

HSV encephalitis is the most common cause of sporadic viral encephalitis and often presents with altered mental status, fever, seizures, and focal neurological deficits [1,2]. In this patient, the diagnosis was confirmed by a positive CSF PCR test for HSV-1. HSV encephalitis can cause significant brain inflammation, particularly affecting the temporal lobes, though in this case, MRI findings revealed only mild chronic microvascular changes and ill-defined hyperintensities in the frontal lobes, presumed to be gliosis [2]. Early treatment with acyclovir is critical in improving outcomes for HSV encephalitis [5].

Neurosyphilis, on the other hand, can manifest with a wide range of neurological symptoms, including cognitive decline, psychiatric symptoms, and movement disorders [3]. The patient's positive rapid plasma reagin (RPR) and CSF VDRL test supported the diagnosis of neurosyphilis [6]. Neurosyphilis can be divided into acute and chronic types. Acute neurosyphilis includes the meningovascular form, which involves inflammation of the meninges and blood vessels and can cause symptoms resembling a stroke along with neurological issues [3]. Chronic neurosyphilis includes conditions like general paresis, which leads to gradual cognitive decline and mental impairment, and tabes dorsalis, which affects the spinal cord and results in sensory loss and coordination problems [6,7]. This classification separates the more immediate inflammatory presentations from the slow, degenerative forms of the disease [6,7].

Autoimmune encephalitis is a rare but serious neurological disorder that can significantly impair individuals, though many cases are treatable. There are several subtypes, each distinguished by the specific antibodies involved. Some forms are associated with antibodies targeting intracellular proteins, while others are linked to antibodies against cell surface antigens or extracellular synaptic proteins [8]. A notable portion of autoimmune encephalitis cases present without detectable antibodies, referred to as "seronegative" autoimmune encephalitis [8]. Certain cases may arise spontaneously, while others are linked to underlying cancers, known as paraneoplastic autoimmune encephalitis [9].

Anti-NMDA receptor (NMDAR) encephalitis, first recognized about 17 years ago, is an autoimmune disorder that presents with rapidly progressive psychiatric symptoms, cognitive decline, seizures, abnormal movements, or coma without a clear cause [10]. Anti-NMDAR encephalitis has an estimated incidence of 1.5 cases per million population per year [4,11]. The disease is marked by the presence of autoantibodies that disrupt NMDAR-mediated synaptic transmission [12]. At the onset of symptoms, differentiating it from primary psychiatric conditions can be difficult, and many patients require intensive care due to the severity of their symptoms. Currently, there are no specific prognostic biomarkers available beyond clinical evaluation. This condition predominantly affects women, with a female-to-male ratio of roughly 8:2, and approximately 37% of patients are under 18 at the time of diagnosis [4]. Known triggers include ovarian teratomas and HSV encephalitis [13,14]. Recent clinical observations indicate that anti-NMDA receptor encephalitis, the most well-known form of antibody-mediated autoimmune encephalitis, may be triggered in some instances by HSV infections [14]. The prognosis is closely tied to the use of aggressive immunotherapy, which frequently includes a combination of corticosteroids, intravenous immunoglobulin, plasma exchange, and, in certain instances, anti-CD20 therapy alongside cyclophosphamide [15].

CJD was also considered in this patient due to the rapid progression of symptoms, elevated 14-3-3 protein levels, and the presence of neurological signs such as tremors and gait disturbances [16]. CJD, a prion disease, typically presents with rapidly progressive dementia, myoclonus, and ataxia and is associated with elevated 14-3-3 protein in the CSF, a marker of neuronal injury [16]. However, in this case, the absence of positive RT-QuIC testing and the lack of characteristic MRI findings made CJD less likely. Additionally, the patient's gradual onset of symptoms over six months is more consistent with infectious and autoimmune causes than the more acute progression seen in CJD. Although elevated 14-3-3 protein was detected, this finding can also occur in other conditions associated with neuronal damage, such as viral encephalitis and autoimmune diseases.

Other neurodegenerative conditions, such as FTD and Alzheimer's disease (AD), were considered, given the significant personality changes and cognitive dysfunction. However, the rapid progression of symptoms, positive infectious and autoimmune markers, and lack of neuroimaging findings typical of these diseases made them less likely. Nonetheless, the patient's progressive dementia and psychiatric symptoms suggest that an underlying neurodegenerative process may be involved, either as a primary condition or secondary to the autoimmune or infectious causes.

Despite prompt treatment with acyclovir and penicillin, the patient's cognitive and functional status remained poor at the time of discharge, with persistent neurological deficits. The long-term prognosis in such cases can be variable and is often dependent on early diagnosis and the extent of brain injury at the time of treatment initiation. Neurological recovery in HSV encephalitis is typically slow, and cognitive impairment may persist, especially in older adults or those with delayed treatment. Similarly, neurosyphilis can result in irreversible neurological damage, even after successful antimicrobial therapy. Repeat imaging and CSF analysis in the coming months will be critical to assess disease progression or resolution. Given the patient's significant functional impairment, further supportive care, including physical rehabilitation and cognitive therapy, may be necessary to optimize recovery. The possibility of a paraneoplastic syndrome, despite negative initial findings, warrants continued surveillance for underlying malignancy.

## Conclusions

This case underscores the complexity of diagnosing and managing patients with rapid cognitive decline and neurological dysfunction, particularly when overlapping infectious and autoimmune processes are involved. A multidisciplinary approach, including neurology, infectious disease, and psychiatry, is essential to address the various facets of such cases and ensure comprehensive care. Neurosyphilis can mimic a range of neurological and psychiatric disorders, including autoimmune encephalitis, making early recognition and timely intervention critical to preventing further cognitive deterioration and morbidity. Additionally, HSV infection has been associated with autoimmune encephalitis, particularly in patients with NMDA receptor positivity. The diagnostic ambiguity seen in this case highlights the limitations of current diagnostic modalities and emphasizes the need for continued research to develop more advanced and precise diagnostic tools.
